# Arterial line pressure control enhanced extracorporeal blood flow prescription in hemodialysis patients

**DOI:** 10.1186/1471-2369-9-15

**Published:** 2008-11-24

**Authors:** Franklin G Mora-Bravo, Alfonso Mariscal, Juan P Herrera–Felix, Salvador Magaña, Guadalupe De-La-Cruz, Nelly Flores, Laura Rosales, Martha Franco, Héctor Pérez-Grovas

**Affiliations:** 1 Department of Nephrology, Instituto Nacional de Cardiología Ignacio Chávez Mexico City, Mexico; 2 Renal Research Institute, New York, New York, USA

## Abstract

**Background:**

In hemodialysis, extracorporeal blood flow (Qb) recommendation is 300–500 mL/min. To achieve the best Qb, we based our prescription on dynamic arterial line pressure (DALP).

**Methods:**

This prospective study included 72 patients with catheter Group 1 (G1), 1877 treatments and 35 arterio-venous (AV) fistulae Group 2 (G2), 1868 treatments. The dialysis staff was trained to prescribe Qb sufficient to obtain DALP between -200 to -250 mmHg. We measured ionic clearance (IK: mL/min), access recirculation, DALP (mmHg) and Qb (mL/min). Six prescription zones were identified: from an optimal A zone (Qb > 400, DALP -200 to -250) to zones with lower Qb E (Qb < 300, DALP -200 to -250) and F (Qb < 300, DALP > -199).

**Results:**

Treatments distribution in A was 695 (37%) in G1 vs. 704 (37.7%) in G2 (*P *= 0.7). In B 150 (8%) in G1 vs. 458 (24.5%) in G2 (*P *< 0.0001). Recirculation in A was 10.0% (Inter quartile rank, IQR 6.5, 14.2) in G1 vs. 9.8% (IQR 7.5, 14.1) in G2 (*P *= 0.62). IK in A was 214 ± 34 (G1) vs. 213 ± 35 (G2) (*P *= 0.65). IK Anova between G2 zones was: A vs. C and D (*P *< 0.000001). Staff prescription adherence was 81.3% (G1) vs. 84.1% (G2) (*P *= 0.02).

**Conclusion:**

In conclusion, an optimal Qb can de prescribed with DALP of -200 mmHg. Staff adherence to DLAP treatment prescription could be reached up to 81.3% in catheters and 84.1% in AV fistulae.

## Background

In dialysis, the clearance directly depends on solute characteristics (small and middle molecular weight), membrane properties (surface area, thickness, efficiency), dialysate flow, and extracorporeal blood flow (Qb) [1,2]. The later is operator dependent, but after selecting the membrane and the dialysate flow rate, Qb prescription is the greatest factor that can be optimized to obtain a greater solute clearance. The Kidney Disease Outcome Quality Initiative (K/DOQI) vascular access recommendations for Qb are between 300 to 500 mL/min [3-5]. This flow is obtained with a peristaltic pump. The pressure generated by the pump carries blood flow into the arterial segment of the dialysis circuit (arterial line) and is measured continuously, denominated dynamic arterial line pressure (DALP). DALP is a negative pressure that has been used to determine catheter dysfunction, which is identified when a dialysis blood flow of 300 mL/min is not being attained in a catheter previously able to deliver greater Qb than 350 mL/min and at a pre pump pressure of -250 mmHg^3^. The instrumentation and continuous measurement of the pressures in the arterial and venous lines allow us to know static and dynamic parameters of the internal pressure in the access site. Pressure measurements have been utilized in the diagnoses of stenosis in the venous side of the access (dynamic and static venous pressure or intra-access pressure), stenosis in the arterial side of the access (static arterial intra-access pressure) or stenosis intra-access (Difference between arterial static pressure and venous static pressure) [6,7]. The study of dynamic venous pressure has demonstrated its usefulness in stenosis detection and thrombosis prevention in grafts [8-13]. Nevertheless these techniques have poor predictive value because the effectiveness to use a ratio of the venous static normalized pressure to the mean arterial pressure in an arterio-venous (AV) graft depends on multiple factors, such as graft location, vascular size, needle location, differentiating between averaged pressures and central pressures. Neither static venous pressure nor dynamic venous pressure has exhibited satisfactory sensitivity or specificity for graft thrombosis [14]. Very little information exists about dynamic arterial line pressure with the exception of its utility in diagnosis of catheter failure. Yet dynamic arterial line pressure can be used for monitoring and optimization of dialysis Qb. K/DOQI research recommendations^3 ^proposed investigations to define the optimum value of Qb, because the range between 300 to 500 ml/min is wide, and an additional need is to reexamine the definition of catheter dysfunction and expand the definition beyond blood flow rates [15]. We propose that the individual prescription of dialysis blood flow, within this wide range of Qb can be optimized for each vascular access based on DALP.

## Methods

### Patients

We enrolled 91 patients from our chronic hemodiafiltration (HDF) dialysis unit and in the renal transplant program from our institute, who received 3 HDF sessions per week (11.5 to 12 hrs/wk). According with their type of vascular access, we divided the study population in two groups: jugular posterior dual-lumen catheters (Group 1) and arterial venous (AV) internal fistulae (Group 2). The dialysis staff was trained to prescribe a Qb enough to obtain a DALP between -200 to -250 mmHg to a maximum Qb goal of 500 ml/min. Patients received anticoagulation with heparin sodium 2.000 units at the beginning of treatment and 1000 units per hour. Additionally, the system filters and lines were heparinised with 1000 units in a dilution of 1 liter of 0.9% saline solution. Institutional policies for patients who are prepared for living donor kidney transplantation undergo dialysis catheters. Patients were dialyzed for only a limited time period (1 to 3 months) as they were waiting for living related kidney transplantation. The hospital usually does not provide tunneled catheters. Patients on the waiting list for cadaveric donors are made arterio venous fistula. The unit of the Institute of Cardiology Ignacio Chavez has just hemodiafiltration machines.

### Treatments

Postdilutional HDF sessions were delivered by volumetric dialysis machines (4008 H; Fresenius Medical Care [FMC], Bad Homburg, Germany). The HDF volume was 17.64 ± 4.9 liters. The machines are equipped with a pre pump measuring system for dynamic arterial line pressure, a blood temperature monitor (BTM) (FMC, Bad Homburg, Germany) for measurements of access recirculation, and an on-line clearance monitor (OCM) (FMC, Bad Homburg, Germany). Polysulfone membrane dialyzers F-60 and F-80 were used (FMC. Walnut Creek, CA). The material of the blood pump segment of the blood line was transparent latex-free rubber for medical use (Fresenius M.C., Fresenius Medical Care AG & Co. KGaA, Bad Homburg, Germany).

Dialysis filters and blood lines were re-used up to 10 ± 4 times and disinfected by formaldehyde after each treatment. Ultra pure bicarbonate dialyzate was delivered. The institutional research committee approved the study. Group 1 had 12-French dual-lumen catheters (Niagara^®^, BARD, Utah, USA). The placement of the catheter was jugular posterior and was performed by the physician of the third year of postgraduate nephrology; the measures were taken for a period of 12 months. In Group 2 we included 19 permanent vascular access points, with native arteriovenous fistulas that were at least 12 weeks old. The anatomic location of the fistulas was, in the lower left arm 14%, in the upper left arm 43%, in the upper left arm 43% and in the upper right arm 43%. This group used 14-French internal diameter needles. Besides the policy of our center, the patients had neither fluid and salt nor dietary restrictions. A high protein and calorie intake (protein catabolic rate >1.4) was recommended and hypertension was well controlled by a strict prescription of dry weight, without antihypertensive medication.

### Data collection

From July 11, 2005 to July 26, 2006, each HDF treatment was recorded in real time and compiled by a telemetry system for dialysis units (Finesse, iSyMed, Butzback, Germany), which provides exportable data to excel software. Multiple information of each treatment was reported and recorded in a database with an algorithm at intervals of 10 minutes. In each session we obtained an average of effective extracorporeal blood flow rate (Qb = mL/min), dynamic arterial line pressure (DALP = mmHg), ionic clearance (IK = mL/min) and access recirculation fraction (%). Dialysis time was not taken into account in the final analysis because the time of dialysis is Variable in each patient. Significant residual renal function needing less dialysis, patients request for earlier termination of the session, nurses' decisions, blood clotting, etc.

### Data analysis

Analysis was performed with a scatter plot. The X axis represented DALP, and Y axis Qb. We divided Qb in high blood flow (> 400 ml/min), middle (300–399 ml/min) and low flow (<300 ml/min). DALP was split in middle arterial line pressure (-249 to -200 mmHg) and low arterial line pressure (<-200 mmHg). For analysis purposes we did not included treatments with high DALP (-300 to -250 mmHg). Within these parameters, 6 prescription quadrants were identified: A zone: Qb > 400 ml/min, DALP -250 to -200 mmHg; B zone: Qb > 400 ml/min, DALP -199 to-100 mmHg; C zone: Qb 300–399 ml/min, DALP -250 to -200 mmHg; D zone: Qb 300–399 ml/min, DALP -199 to -100 mmHg and E zone: Qb < 300 ml/min, DALP -250 to -200 mmHg and F Zone: Qb < 300 ml/min, DALP -199 to -100 mmHg. We compared staff prescription adherence, ionic clearance and recirculation fraction between the two groups of study. Additionally we compared ionic clearance between zones in Group 2.

### Statistical analysis

Parametric data distribution was expressed as mean and standard deviation, and non parametric as median and Inter Quartile Rank (IQR) 25–75. Test for Normality was performed with Shapiro-Wilk test. Statistical analysis was evaluated by T test, ANOVA and Wilcoxon's signed rank test according to normality and grouping. *P *values of ≤ 0.05 were considered statistically significant. SPSS for Windows 13.0 software (SSPS, Inc., Chicago, IL) was used for all statistical analysis.

## Results

There were 91 patients, 72 in Group 1 with vascular access by means of jugular catheter and 19 in Group 2 with AV fistulae. 35 females in Group 1, and 9 in Group 2. Mean age in Group 1 was 33.4 ± 15 vs 44.5 ± 16 in Group 2. There was no evidence of cardiac dysfunction with ejection fraction of 58.6 ± 9.6% in Group 1 vs. 59.3 ± 4.8% (p = 0.68) in Group 2, by the Simpson method of Echocardiography.

Previous to kidney transplantation, all patients in Group 1 were waiting for living donors (100%) and in Group 2 for cadaveric donors. No antihypertensive drugs were used, intravenous iron was administered in 38 patients (4 in Group 2), erythropoietin was required in 6 patients (1 in Group 2) and 2 patients in Group 2 received vitamin D analogues. We did not have vascular access failure in Group 2 during the trial time.

We recorded 1877 treatments in Group 1 (20 treatments/patient) and 1868 treatments in Group 2 (98 treatments/patient). The distribution of treatments according to the identified zone were as follows; in Group 1: A zone 695 (37%), B zone 150 (8%), C zone 604 (32.2%), D zone 272 (14.5%), E zone 77 (4.1%) and F zone 79 (4.2%) (*P *< 0.000001), in Group 2: A zone 704 (37.7%), B zone 458 (24.5%), C zone 390 (21.0%), D zone 247 (13.2%), E zone 19 (1%) and F zone 50 (2.6%) (*P *< 0.000001). Comparisons between both groups are represented in table 1. 103 measures with DALP < -250 mmHg were recorded in group 1 and 57 in group 2.

**Table 1:** Treatments distribution between zones, for catheters (group 1) and AV fistulae (group 2)

Zones	Group 1 n	%	Group 2 N	%	*P-value*
A zone	695	37.0	704	37.7	0.70
B zone	150	8.0	458	24.5	<0.0001
C zone	604	32.2	390	21.0	<0.0001
D zone	272	14.5	247	13.2	0.28
E zone	77	4.1	19	1.0	<0.0001
F zone	79	4.2	50	2.6	0.01

Total	1877	100	1868	100	

**Figure 1:**
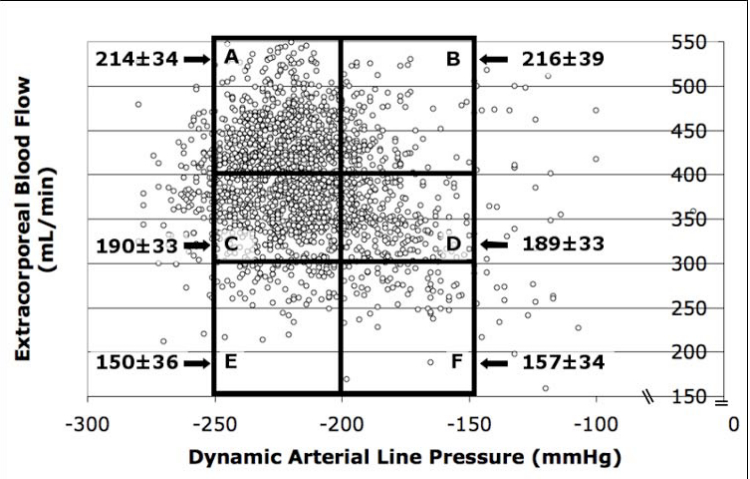
**Scatter-plot and prescription zones in catheters (group 1)**. Scatter-plot and prescription zones in Group 1: Y Axis Extracorporeal blood flow (Qb) (mL/min). X axis Dynamic Arterial Line Pressure (DALP) (mmHg). A Zone: Qb > 400 ml/min, DALP -250 to -200 mmHg; B Zone: Qb > 400 ml/min, DALP-199 to -100 mmHg; C Zone: Qb 300–399 ml/min, DALP -250 to -200 mmHg; D zone: Qb 300–399 ml/min, DALP-199 to -100 mmHg; E Zone: Qb < 300 ml/min, DALP -250 to -200 mmHg; F Zone: QS < 300 ml/min, DALP -199 to -100 mmHg. Ionic clearance value is shown in each zone (mL/min) with arrows.

**Figure 2:**
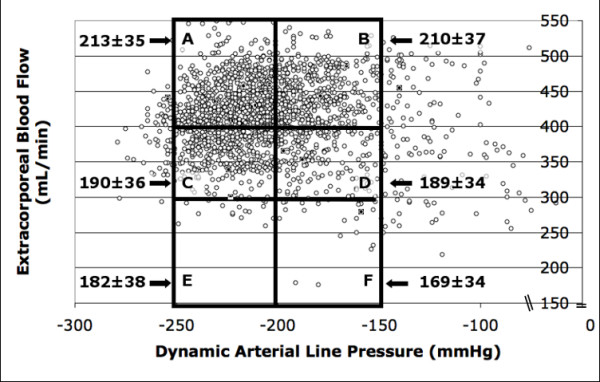
**Scatter-plot and prescription zones in AV fistulae (group 2)**. Scatter-plot and prescription zones in Group 2. Y Axis Extracorporeal blood flow (Qb) (mL/min). X axis Dynamic Arterial Line Pressure (DALP) (mmHg). A Zone: Qb > 400 ml/min, DALP -250 to -200 mmHg; B Zone: Qb > 400 ml/min, DALP-199 to -100 mmHg; C Zone: Qb 300–399 ml/min, DALP -250 to -200 mmHg; D zone: Qb 300–399 ml/min, DALP-199 to -100 mmHg; E Zone: Qb < 300 ml/min, DALP -250 to -200 mmHg; F Zone: QS < 300 ml/min, DALP -199 to -100 mmHg. Ionic clearance is shown in each zone (mL/min) with arrows. Ionic clearance value is shown in each zone (mL/min) with arrows.

A to F zones correspond to treatments distribution in Figure 1 and Figure 2, n, number of treatments; %, percentage of treatments.

Staff adherence to Qb and DALP prescription in A, B, C and D zones in Group 1 was 1526 treatments 81.3%, while in Group 2 was 1571 treatments 84.1% (*P *= 0.02).

Dialysis time in Group 1 was different in each zone with a range from 217 ± 26 minutes in E zone to 229 ± 25 minutes in D zone. Qb in Group 1, A zone was 448 ± 35 ml/min vs. 360 ± 26 mL/min (*P *< 0.000001) in C zone. Qb Anova between zones in Group 1 was *P *< 0.000001. (Table 2). DALP in Group 1 was similar in zone A, C and E (*P*: NS). A zone vs B, D and F zones (*P *< 0.000001).

**Table 2:** Characteristics of time, Qb and DALP in catheters (group 1)

	A zone	B zone	C zone	D zone	E zone	F zone	*P-value*
Time (min)	220 ± 24	228 ± 25	223 ± 25	229 ± 25	217 ± 26	223 ± 28	<0.000001
Qb (ml/min)	448 ± 35*	448 ± 36	360 ± 26*	350 ± 27*	276 ± 20	264 ± 28	<0.000001
DALP (mmHg)	-224 ± 13**	-180 ± 22**	-223 ± 14	-181 ± 20**	-222 ± 13	-167 ± 23**	<0.000001

Time: length of treatment; Qb, extracorporeal blood flow; DALP dynamic arterial line pressure; A to F zones correspond to treatment distribution in Figures 1 and 2; *P > 0.000001 Qb between, A vs C and A vs D zones; **P < 0.000001 DALP A vs B and D zones and F zones.

Dialysis time in Group 2 was different in each zone with a range from 224 ± 24 minutes in F zone to 234 ± 20 minutes in C zone. Qb in Group 2 A zone was 442 ± 32 ml/min vs. 369 ± 24 mL/min in C zone (*P *< 0.000001). Qb Anova between zones in Group 2 was *P *< 0.000001. (Table 3). Dynamic arterial line pressure in Group 2 was similar in zone A, C and E (*P*: NS), while there was a significant difference in A zone vs B, D and F zones (*P *< 0.000001).

**Table 3:** Characteristics of time, Qb and DALP in AV fistulae (group 2)

	A zone	B zone	C zone	D zone	E zone	F zone	*P-value*
Time (min)	232 ± 19	231 ± 20	234 ± 20	230 ± 22	227 ± 18	224 ± 24	0.007
Qb (Ml/min)	442 ± 32*	452 ± 32	369 ± 24*	*361 ± 29	283 ± 12	270 ± 34	<0.000001
DALP (mmHg)	-221 ± 13**	-174 ± 24**	-224 ± 13	-167 ± 30**	-220 ± 13	-143 ± 40**	<0.000001

Time, length of treatment; Qb, extracorporeal blood flow; DALP, dynamic arterial line pressure; *P < 0.000001, Qb A vs. C and A vs. D zone; **P < 0.000001; DALP, A vs. B, D and F zones; *P*: NS, not significant, DALP A vs. C and E zones

Recirculation fraction in A zone was 10.0% (IQR 6.5 – 14.2) in Group 1 vs. 9.8% (IQR 7.5 – 14.1) in Group 2 (*P *= 0.62). (Table 4). Recirculation in F zone was 14.5 (IQR 5.7 – 17.5) in Group 1 vs. 12.5 (IQR 7.5 – 19.2) in Group 2 (*P *= 0.83).

**Table 4:** Access recirculation for catheter (group 1) and AV fistulae (group 2)

Zones	Group 1 Recirculation (%)	IQR	Group 2 Recirculation (%)	IQR	*P-value*
A zone	10.0	6.5 – 14.2	9.8	7.5 – 14.1	0.62
B zone	9.6	6.1 – 15.5	12.2	8.5 – 14.4	0.07
C zone	12.6	8.2 – 17.9	10.7	7.6 – 16.5	0.21
D zone	9.3	6.2 – 15.0	10.0	7.5 – 12.6	0.69
E zone	10.9	6.4 – 26.0	6.6	6.5 – 6.6	0.77
F zone	14.5	5.7 – 17.5	12.5	7.5 – 19.2	0.83

Recirculation: Access recirculation fraction; IQR, Interquartile range. A to F zones correspond to treatment distribution in Figures 1 and 2.

Ionic clearance in Group 1 was different in each zone in a range from 214 ± 34 mL/min in A zone to 157 ± 34 mL/min in F zone. Comparison of ionic clearance in A zone vs B zone (216 ± 39 mL/min) was no significant, but when comparing C zone (190 ± 33 mL/min) and D zone (189 ± 33 mL/min) to A, B, E and F zones was significant *P *< 0.000001 (Figure 1, Table 5).

**Table 5:** Ionic clearance in catheter and AV fistulae

	A zone	B zone	C zone	D zone	E zone	F zone	**P-value
Group 1	214 ± 34	216 ± 39	190 ± 33	189 ± 33	150 ± 36	157 ± 34	<0.000001
Group 2	213 ± 35*	210 ± 37	190 ± 36	189 ± 34*	182 ± 38	169 ± 34	<0.000001
*P-value	0.6	0.65	0.9	0.44	0.001	0.48	

Group 1, Ionic clearance (mL/min) in catheters; Group 2, Ionic clearance in AV Fistulas; *P < 0.001 T test; **P < 0.0001; Anova test; A to F zones correspond to treatment distribution in Figures 1 and 2.

Ionic clearance in Group 2 was different in each zone in a range from 213 ± 35 mL/min in A zone to 169 ± 34 mL/min in F zone. Anova between zones in Group 2 ionic clearance was *P *< 0.000001 when comparing A zone vs C (190 ± 33 mL/min) and A vs D (189 ± 34 mL/min) zones (Figure 2, Table 5). Ionic clearance in A zone vs B zone (210 ± 37 mL/min) was not statistically significant.

T test for ionic clearance performed in the same treatment zones comparing Group 1 versus Group 2 was no significant, with the exception of E zone, where greater ionic clearance was found in Group 2 compared to Group 1 (182 ± 38 mL/min vs, 150 ± 36 mL/min respectively *P *= 0.001). There was no difference in ionic clearances between zones that corresponded to an equivalent Qb, such as A and B; C and D; and E an F (*P *= NS), in Grup1 as well as in Group 2 (Table 5).

## Discussion

**Figure 3:**
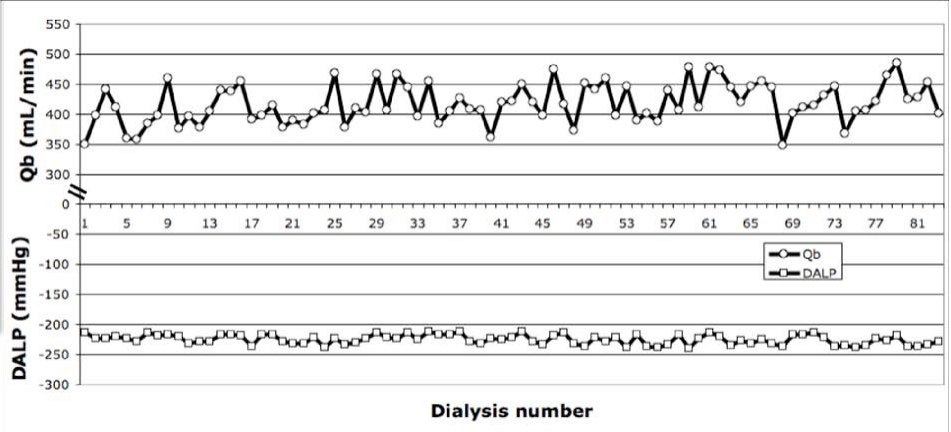
**Progression of DALP and Qb in a patient with AV fistulae during 83 HDF sessions**. Representative illustration during 83 hemodiafiltration treatments: 3 times per week in a patient with AV fistulae access before renal transplant. Each point represents the average on an entire procedure of dynamic arterial line pressure (DALP) and Extracorporeal blood flow (Qb). Observe the tendency for prescription based on DALP (between -250 to -200 mmHg) with different extracorporeal blood flow for each treatment.

Dynamic arterial line pressure is a negative pressure generated by the machine's peristaltic pump. The dynamic nomination must be, so that it translates the negative pressure of the pump in movement from arterial line to vascular access. This study describes extracorporeal blood flow prescription based on DALP which permits to optimize Qb until limits near to 500 ml/min could be attained. The best extracorporeal blood flow (>400 ml/min) (A and B zones) was achieved in higher percentage in patients with AV fistulae accesses 62.2%, compared to 45% in the catheter group. On the other hand the lower or worse extracorporeal blood flow in zones E and F (<300 mL/min) was found in higher percentage in the catheter group 8.3%, while in the fistulae group was 3.6%. This is evidence that a better extracorporeal blow flow from what it is currently recommended could be obtained with AV internal fistulae. Contrary to what is usually expected at higher blood flows, the recirculation fraction was not modified in any zone, this means that the negative pressure in the arterial line between -200 to -250 mmHg did not affect the recirculation fraction, and even though we did not measure access blood flow, the unchanged recirculation fraction could be due to well constructed vascular accesses. Dynamic arterial line pressure had an average of -222 mmHg in A, C and D zones (Group 1 and Group2) with the best extracorporeal blood flow in each zone. DALP parameters could be used to formulate diagnosis of access quality and help to monitor extracorporeal blood flow over time (intradialysis and interdialysis) (See figure 3). Therefore DALP parameters could be helpful identifying catheter and AV fistulae dysfunction. Vascular access dysfunction which is defined when an extracorporeal blood flow is below 300 mL/min at a pre pump pressure of -220 mmHg, taken with Qb cut point 300 mL/min as low extracorporeal blood flow based on K/DOQI recommendation^3^. A zone with the major blood flow and an optimal DALP would be the aimed zone in each treatment. B zone is a special zone, where the excellent quality of the access apparently does not require prescription based on DALP. C zone is a middle flow zone that nevertheless has an optimized prescription. Zone D is a zone that needs to be optimized, where greater emphasis in obtaining an increase in the extracorporeal flow can be placed. Probably with an increase of DALP this treatment zone and/or most of the treatments would be located in A zone. D zone in higher percentage was identified as the zone where staff failure to prescription attachment occurred. E zone consisted of accesses with low blood flow even though DALP was optimal. Nevertheless with an optimized prescription E zone in AV fistulas could correspond to immature accesses. F zone consisted of accesses of poor quality superposed to a non-optimal treatment prescription, probably if we optimize DALP we could attain a prescription reaching C zone. Ionic clearance in zones that corresponded to an equivalent extracorporeal blood flow was no different, however it is interesting to note that as mentioned previously the procedures located in D zone, with ionic clearances of 189 ± 34 could be improved with a better prescription of DALP. The enhanced prescription could explain the higher ionic clearance of 214+34 mL/min observed in A zone.

Overall, the proposed policy to evaluate DALP and to aim for maximum Qb is well established in most HD units with properly trained nursing staff. The main problem is when these targets (high Qb with acceptably low DALP) can not be achieved.

As there was no statistical significance found in clearance between A and B categories both in group 1 and group 2, the final recommendation for using QB in excess of 400 ml/min should not say that those QBs should be attempted only when DALP does not exceed -200 mmHg.

Otherwise, we are just providing confirmatory data that dialysis catheters are not as good as a native A-V fistula for the average patient in the short or long run after catheter insertion. QB should in some way correspond to the dialyser size chosen. High QB would be of no effect in a low surface area dialysers and vice versa low QB would cause coagulation problems in large area dialyser types.

Disadvantages of our study were non-measured access blood flow and no available information on pain evaluation during needle insertion. We do not have kt/v with three samples, nevertheless, ionic dialysance and urea clearance proved to be closely correlated (r^2 ^= 0.89) [16]. Another disadvantage is we do not measure LDH and free hemoglobin in patients at high pressures lower than -250 mmHg to diagnose hemolysis.

Our study describes a novel concept of optimizing dialysis treatment prescription based on DALP identified zones, that could be used in the future for a feedback system on extracorporeal blood flow, by increasing or diminishing intradialysis Qb guided by DALP (See figure 3).

### Conclusion

In conclusion this investigation has shown that Qb prescription can be optimized by DALP. DALP of -200 mmHg is recommended for obtaining the best Qb. Staff adherence to DLAP treatment prescription could be reached up to 81.3% in catheters and 84.1% in AV fistulae.

## Competing interests

The authors declare that they have no competing interests.

## Authors' contributions

FM, HPG and LAM participated in the design of the study and performed the statistical analysis. GDC, NF, JPH, SM carried out the record of patients. MFG, LR conceived of the study, and participated in its design and coordination and helped to draft the manuscript. All authors read and approved the final manuscript.

## Pre-publication history

The pre-publication history for this paper can be accessed here:



## References

[B1] Daugirdas JT (1993). Second generation logarithmic estimates of single-pool variable volume Kt/V: An analysis of error. J Am Soc Nephrol.

[B2] Gotch FA, Sargent JA (1985). A mechanistic analysis of the National Cooperative Dialysis Study (NCDS). Kidney Int.

[B3] Schwab S, Besarab A, Beathard G, Brouwer D, Etheredge E, Hartigan M, Levine M, McCann R, Sherman R, Trertola S, K/DOQI Vascular Access Work Group Recommendations for clinical practice guidelinesfor vascular access. [National Kidney Foundation K/DOQI web site]. http://www.kidney.org/professionals/kdoqi/guidelines_updates/doqiupva_ii.html#doqiupva10.

[B4] Besarab A, Ross R, Al-Aljel F, Deane C, Frinak S, Zasuwa G (1995). The relation of brachial artery flow to access flow. J Am Soc Nephrol.

[B5] Besarab A, Sherman RA (1997). The relationship of recirculation to access blood type. Am J Kidney Dis.

[B6] Sullivan KL, Besarab A (1997). Hemodynamic screening and early percutaneous intervention reduce hemodialysis access thrombosis and increase graft longevity. J Vasc Intervent Radiol.

[B7] Turnel-Rodrigues L, Pengloan J, Baudin S, Testou D, Abaza M, Dahdah G, Mouton A, Blanchard D (2000). Treatment of stenosis and thrombosis in hemodialysis fistulas and grafts by interventional radiology. Nephrol dial Transplant.

[B8] Krivitski NikolaiM (2003). Access Flow Measurement During Surveillance and Percutaneous transluminal Angioplasty Intervention. Seminars in Dialysis.

[B9] Schwab SJ, Raymond JR, Saeed M, Newman GE, Dennis PA, Bollinger RR (1989). Prevention of hemodialysis fistula thrombosis. Early detection of venous stenosis. Kidney Int.

[B10] Cayco AV, Abu-Alfa AK, Mahnensmith RL, Perazzella MA (1998). Reduction in arteriovenous graft impairment: results of a vascular access surveillance protocol. Am J Kidney Dis.

[B11] Safa AA, Valji K, Roberts AC, Ziegler TM, Hye RJ, Oglevie SB (1996). Detection and treatment of dysfunctional hemodialysis access grafts: Effect of a surveillance program on graft latency and the incidence of thrombosis. Radiology.

[B12] Greenwood RN, Aldridge C, Goldstein L, Baker LR, Cattell WR (1985). Assessment of arteriovenous fistulae from pressure and thermal dilution studies: clinical experience in forearm fistulae. Clin Nephrol.

[B13] Smits JH, Linden J Van der, Hagen EC, Modderkolk-Cammeraat EC, Feith GW, Koomans HA, Dorpel MA Van den, Blankestijn PJ (2001). Graft surveillance: venous pressure, access flow, or the combination?. Kidney Int.

[B14] Singh N, Ahmad S, Wienckowski JR, Murray BM (2006). Comparison of access blood flow and venous pressure measurements as predictors of arteriovenous graft thrombosis. J Vasc Access.

[B15] Moist LM, Hemmelgarnand BR, Lok CE (2006). Relationship between Blood Flow in Central Venous Catheters and Hemodialysis Adequacy. Clin J Am Soc Nephrol.

[B16] Manzoni C, Di Filippo S, Corti M, Locatelli F (1996). Ionic dialisance as a method for the on-line monitoring of delivered dialysis without blood sampling. Nephrol Dial Transplant.

